# Influence of Apnea Hypopnea Index and the Degree of Airflow Limitation on Endothelial Function in Patients Undergoing Diagnostic Coronary Angiography

**DOI:** 10.3390/biology11030457

**Published:** 2022-03-17

**Authors:** Dorota Ochijewicz, Adam Rdzanek, Tadeusz Przybyłowski, Renata Rubinsztajn, Monika Budnik, Ewa Pędzich, Katarzyna Białek-Gosk, Piotr Bielicki, Agnieszka Kapłon-Cieślicka

**Affiliations:** 1 1st Department of Cardiology, Medical University of Warsaw, 02-091 Warsaw, Poland; dochijewicz@wum.edu.pl (D.O.); monika.budnik@wum.edu.pl (M.B.); ewa.pedzich@wp.pl (E.P.); agnieszka.kaplon-cieslicka@wum.edu.pl (A.K.-C.); 2 Department of Internal Medicine, Pulmonary Diseases and Allergy, Medical University of Warsaw, 02-091 Warsaw, Poland; tadeusz.przybylowski@wum.edu.pl (T.P.); rrubinsztajn@wum.edu.pl (R.R.); katarzyna.bialek-gosk@wum.edu.pl (K.B.-G.); piotr.bielicki@wum.edu.pl (P.B.)

**Keywords:** obstructive sleep apnea, endothelial dysfunction, chronic coronary syndrome

## Abstract

**Simple Summary:**

Obstructive sleep apnea and airflow limitation disorders are linked to increased cardiovascular morbidity and mortality in patients with chronic coronary syndrome. Although the exact mechanism associated with this phenomenon remains poorly elucidated, the impairment of endothelial function observed in both breathing disorders and cardiovascular disease is one of the possible pathophysiological processes linking those conditions. In the present study, we sought to determine the possible relationship between the endothelial function, signs of disturbed respiration during sleep, and airflow limitation in chronic coronary syndrome patients undergoing diagnostic invasive coronary angiography. Our study showed that obstructive sleep apnea signs measured by WatchPAT (respiratory disturbance index, apnea and hypopnea index, and oxygen desaturation index) were associated with endothelial dysfunction. Additionally, greater airflow limitation by spirometry was detected in patients with endothelial dysfunction. Patients with endothelial dysfunction showed an increase in left ventricular hypertrophy with a trend of increase in left atrial enlargement, indicating underlying diastolic dysfunction. However, the endothelial dysfunction was independent of the presence or severity of obstructive coronary artery disease in coronary angiography. We believe that our study may complement and extend the current understanding of endothelial dysfunction in the mechanism, explaining the relationship between sleep apnea and cardiovascular diseases.

**Abstract:**

Background: Obstructive sleep apnea is associated with an increased prevalence of cardiovascular disease. The mechanism of these associations is not completely understood. We aimed to investigate the association of the apnea hypopnea index and the degree of airflow limitation with endothelial dysfunction. Methods: This was a single-center prospective study of patients admitted for diagnostic coronary angiography (CAG). Endothelial function was assessed by the non-invasive EndoPAT system by reactive hyperemia index (RHI) and divided into two groups: endothelial dysfunction and normal endothelial function. Sleep apnea signs were detected by WatchPAT measuring the respiratory disturbance index (pRDI), the apnea and hypopnea index (pAHI), and the oxygen desaturation index (ODI). Patients underwent spirometry and body plethysmography. Based on CAG, the severity of coronary artery disease was assessed as follows: no significant coronary artery disease, single-, two- and three-vessel disease. Results: A total of 113 patients were included in the study. Breathing disorders measured by WatchPAT and spirometry were more severe in patients with endothelial dysfunction: pRDI (27.3 vs. 14.8, *p* = 0.001), pAHI (24.6 vs. 10.3, *p* < 0.001), ODI (13.7 vs. 5.2, *p* = 0.002), forced expiratory volume in one second (FEV_1_) (81.2 vs. 89, *p* = 0.05). In a multivariate regression analysis, pAHI and FEV_1_ were independent predictors of endothelial dysfunction assessed by RHI. There was no correlation between the severity of coronary artery disease and endothelial dysfunction. Conclusions: Obstructive sleep apnea signs and greater airflow limitation were associated with endothelial dysfunction regardless of the severity of the coronary artery disease.

## 1. Introduction

Obstructive sleep apnea (OSA) and airflow limitation disorders are highly prevalent in patients with chronic coronary syndrome (CCS) [1,2,3]. Intermittent hypoxia associated with disordered breathing events may lead to oxidative stress, sympathetic activation, inflammation and endothelial dysfunction, all of which are relevant mediators of cardiovascular diseases. Although the exact mechanism associated with this phenomenon remains poorly elucidated, the impairment of endothelial function observed in both breathing disorders and cardiovascular diseases is one of the possible pathophysiological processes linking those conditions [4]. Apart from endothelial dysfunction, the severity of OSA has been independently linked to the presence of coronary plaques and coronary plaque burden assessed by coronary computed tomographic angiography [5]. Whether the association of sleep breathing disorders with endothelial dysfunction varies by the severity of atherosclerosis assessed by coronary angiography (CAG) remains unknown. Accordingly, in the present study, we sought to determine the possible relationship between the endothelial function, signs of disturbed respiration during sleep, and airflow limitation in chronic coronary syndrome patients undergoing diagnostic invasive angiography.

## 2. Materials and Methods

In this single-center prospective study, consecutive patients admitted for diagnostic CAG were included. The inclusion criteria were indications for CAG based on clinical judgment or non-invasive testing, age >18 years old, and the ability to read, understand and sign the informed consent form. The exclusion criteria were a history of a recent (less than 30 days) acute coronary syndrome, severe valvular heart disease, the coexistence of a chronic disease that significantly limits the expected survival time of the patient, contraindications to perform spirometry, a lack of informed consent to participate in the study, recent acute heart failure, and a known malignancy or coexisting systemic inflammatory disease. All subjects underwent standard evaluation by means of medical history, clinical examination, laboratory measurements, electrocardiogram and echocardiography. The presence of comorbidities was obtained from each of the patients by a trained physician upon the medical history. Based on the CAG, patients were classified into four categories: no significant coronary artery disease, single-, two- and three-vessel disease. No significant coronary artery disease was diagnosed according to the standard definition of non-obstructive coronary artery disease (angiographic stenosis of less than 50%). Endothelial function was assessed by the non-invasive EndoPAT system (Itamar Medical Ltd., Caesarea, Israel) based on the peripheral arterial tonometry (PAT)—plethysmography signal technology that measures endothelium-mediated changes in vascular tone via biosensors placed on the fingertips. The endothelium-mediated changes were obtained by a 5-min occlusion of the brachial artery using a standard blood pressure cuff inflated to supra-systolic pressure. The endothelium-dependent flow-mediated dilatation was assessed by the reactive hyperemia index (RHI). The contra-lateral arm, with simultaneous readings from the index fingers, was used as the control. Endothelial dysfunction (ED) was diagnosed in patients with an RHI less than 1.67 [6].

Sleeping disorders were screened for OSA symptoms with a WatchPAT examination (WatchPAT 200 Itamar Medical Ltd., Caesarea, Israel) during a hospital stay. The WatchPAT is a sleep data recorder connected to a finger probe with a plethysmograph and a pulse oximeter. Clinical parameters, such as the PAT respiratory disturbance index (pRDI), PAT apnea and hypopnea index (pAHI), and a 4% oxygen desaturation index (ODI) were analyzed by the WatchPAT proprietary software algorithm. All patients completed the Epworth sleepiness scale (ESS), and the chronic obstructive pulmonary disease assessment test (CAT). All patients underwent extended pulmonary function tests with spirometry and lung plethysmography according to the standard protocol [7,8].

The study was conducted in accordance with the Declaration of Helsinki of the World Medical Association. The Medical University of Warsaw ethics committee approved the study protocol, and written informed consent was provided by all the patients.

### Statistical Analysis

Continuous variables with non-normal distribution were presented as the median and interquartile ranges (IQR). Continuous variables with normal distribution were presented as the mean value ± standard deviation. The type of distribution was assessed using the Kolmogorov-Smirnov test. Categorical variables were presented as absolute values and percentages. Differences between non-normally and normally distributed continuous variables were calculated with the Mann-Whitney U test and Student’s *t* test, respectively. Differences between categorical variables were calculated with Fisher’s exact test. Continuous related variables were compared by the paired Student’s t test or Wilcoxon signed-rank tests, and categorical related variables were compared using the McNemar test. Multivariate regression analysis was performed to determine the factors influencing the reactive hyperemia index. Correlations between variables were analyzed with Pearson’s or Spearman’s coefficient, depending on the data distribution. All statistical tests were two-sided and the *p* value < 0.05 was considered statistically significant. Statistical analysis was performed using IBM SPSS Statistics (version 26).

## 3. Results

**Figure 1:**
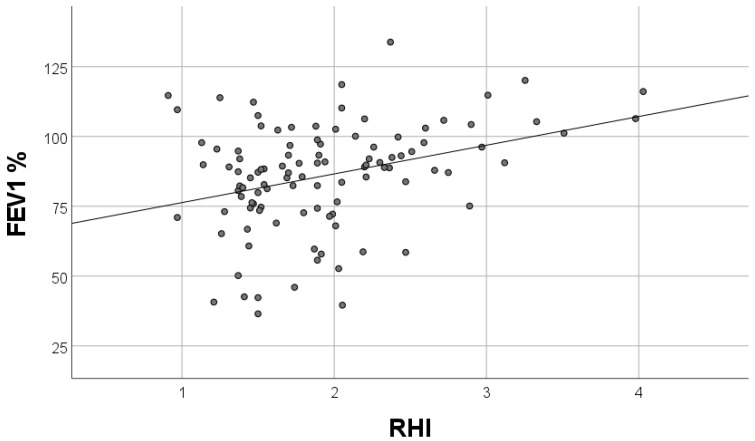
Correlation of forced expiratory volume in one second (FEV1%) and reactive hyperemia index values (RHI) R = 0.331; *p* = 0.001.

**Figure 2:**
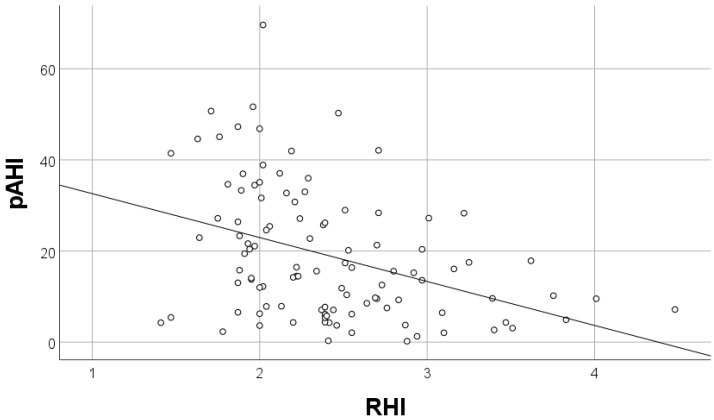
Correlation of peripheral artery tone acquired apnea/hypopnea index (pAHI) and reactive hyperemia index (RHI) values R= −0.387; *p* < 0.001.

**Figure 3:**
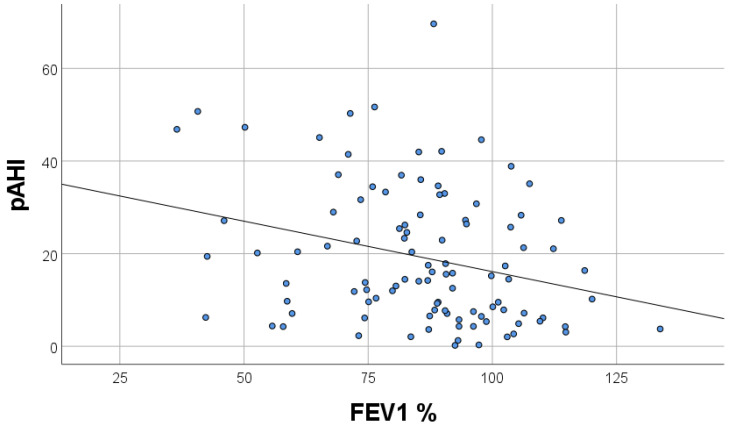
Correlation of baseline forced expiratory volume in one second (FEV1%) and peripheral artery tone acquired apnea/hypopnea index (pAHI) R= −0.280; *p* = 0.004.

A total of 113 patients met the inclusion criteria. Baseline clinical characteristics of patients with and without endothelial dysfunction are summarized in Table 1. No significant differences were recorded, except for higher body mass index (BMI) (30.5 vs. no-NA: 28.5 kg/m^2^, *p* = 0.049) and a higher frequency of loop diuretics administration (15% vs. 8%, *p* = 0.003) in patients with endothelial dysfunction. The presence and severity of coronary artery disease were similar in both groups (Table 2). Patients with endothelial dysfunction had a greater left atrium diameter and left ventricle wall thickness (Table 2). Sleep breathing disorders, measured by WatchPAT, were more severe in patients with endothelial dysfunction that is pRDI (27.3 vs. 14.8, *p* = 0.001), pAHI (24.6 vs. 10.3, *p* < 0.001), and ODI (13.7 vs. 5.2, *p* = 0.002) (Table 3). Airflow limitation by spirometry was more severe in patients with endothelial dysfunction (forced expiratory volume in one second (FEV_1_) (81.2 vs. 89, *p* = 0.05)). In the multivariate regression analysis, pAHI and FEV_1_ were independent factors of endothelial dysfunction, assessed by RHI (Table 4). FEV_1_ had a positive correlation and pAHI had a negative correlation with RHI (Figure 1 and Figure 2). Furthermore, pAHI had a weak negative correlation with FEV_1_ (Figure 3).

## 4. Discussion

This prospective registry of patients admitted for the diagnostic CAG showed that obstructive sleep apnea signs measured by WatchPAT: pRDI, pAHI and ODI were associated with endothelial dysfunction. Additionally, greater airflow limitation by spirometry was detected in patients with endothelial dysfunction. Patients with endothelial dysfunction showed an increase in left ventricular hypertrophy with a trend of increase in left atrial enlargement, indicating underlying diastolic dysfunction. However, the endothelial dysfunction was independent of the presence or severity of obstructive coronary artery disease in CAG. In the current study, pAHI during sleep was inversely correlated with RHI, indicating that greater pAHI during sleep was associated with slower arterial response after ischemia.

Endothelial cells produce nitric oxide, promote local vasodilatation, and inhibit platelet aggregation, monocyte adhesion and vascular smooth muscle proliferation [9]. As a consequence, endothelial dysfunction is often considered one of the earliest detectable and possibly reversible abnormalities in the development of atherosclerosis [3,10]. Emerging studies indicate that in obstructive sleep apnea, intermittent hypoxia, fluctuations in the partial pressure of carbon dioxide, negative intrathoracic pressure swings, decreased parasympathetic and increased sympathetic activity can lead to endothelial dysfunction, but the exact pathophysiological mechanism is not well understood [11,12,13,14]. It has been suggested that intermittent hypoxemia during sleep increases oxidative stress, the production of free radicals. This may cause an inflammatory cascade, leading to increased vascular permeability, vasoconstriction, a proliferation of vascular smooth muscle cells, platelet aggregation, thrombosis and the acceleration of atherosclerosis [4,15,16]. Intermittent hypoxia triggers mitochondrial dysfunction, resulting in increased upregulation of nuclear factor kappa B (NF-κB) in neutrophils/monocytes, increasing the production of adhesion molecules, inflammatory cytokines and adipokines [17]. Vascular wall hypoxia promotes the thrombogenic potential of atherosclerotic plaques and thrombus formation via prothrombotic factor upregulation [17]. Skin biopsies obtained from OSA patients demonstrate a significant upregulation of endothelial nitric oxide synthase (eNOS), tumor necrosis factor-induced protein 3, hypoxia-inducible factor 1 alpha (HIF-1α), vascular endothelial growth factor (VEGF), and vascular cell adhesion molecule 1 (VCAM-1) [18].The multifactorial association of OSA and cardiovascular diseases has been proposed in the meta-analysis conducted by Wang et al. Moderate/severe OSA reduced endothelial function, increased arterial stiffness, and caused chronic inflammation in patients without overt cardiovascular disease or diabetes [15].

Treatment of OSA with continuous positive airway pressure (CPAP) has improved endothelial dysfunction, insulin sensitivity, decreased systemic blood pressure, pulmonary artery pressure, and reduced atrial fibrillation risk [13,19,20,21,22,23,24,25]. However, well-conducted randomized clinical trials did not show a reduction in major cardiovascular events [25,26]. The benefit was observed when CPAP therapy was used for at least 4 h per night [27,28]. The issue of poor compliance is a major limitation of CPAP trials.

Our results, suggesting that OSA is associated with endothelial dysfunction, are consistent with prior studies. Shpilsky et al. have demonstrated that moderate/severe OSA was modestly associated with endothelial dysfunction, determined by RHI and subclinical atherosclerotic coronary artery disease, measured by coronary artery calcium [11]. In a large community-based sample of generally healthy older individuals from the Sleep Heart Health Study (SHHS), sleep apnea was associated with endothelial dysfunction, which was determined by brachial artery flow-mediated dilation (FMD) [29]. Among individuals with a high cardiovascular risk or with established cardiovascular disease, Seif et al. have shown that moderate to severe intermittent hypoxia (defined by ODI) is associated with decrements in endothelial function [30]. The results of our study, and of previous meta-analyses, suggest that the severity of OSA may correlate with endothelial dysfunction and is not modified by potential covariates, such as BMI [15,31].

Obesity is the major risk factor for OSA, mostly due to direct mechanical effects on the respiratory system [32,33]. In our study, higher BMI was associated with endothelial dysfunction and could be one of the leading causes of OSA. Obesity may magnify the effects of OSA in promoting a progressive inflammatory state. Combined therapy with CPAP and weight-loss intervention resulted in a greater reduction in blood pressure, as compared with either intervention alone [34]. Another risk factor of sleep apnea that has been proposed recently, was an excess of fluid around the upper airway in patients with heart failure [35]. A redistribution of fluid in individuals with lower extremity edema, when supine, may lead to the collapse of the upper airway during sleep [4]. Therapeutic approaches like the use of diuretics and exercises have been shown to improve symptoms of sleep apnea [36]. However, patients with heart failure experience the coexistence of both obstructive and central sleep apnea (due to an increased pulmonary capillary pressure) [4]. Recent studies have shown that endothelial function is also impaired in patients with chronic obstructive pulmonary disease (COPD) and the level of impairment is correlated with the severity of airway obstruction [37]. Furthermore, increased endothelial permeability, infiltration of neutrophils, and intimal hyperplasia were involved in the pathogenesis of vascular remodeling during the early-stage of COPD [38,39,40]. In our study, in patients with no overt COPD, a greater airflow limitation was associated with endothelial dysfunction and could be a prognostic factor of high cardiovascular risk.

### Study Limitations

This is a small, single-center study. Selection bias cannot be excluded. We have screened sleep disorders using the WatchPAT system. Polysomnography may have provided more detailed sleep characteristics for analysis. The clinical relevance of our results is still unclear, warranting future studies with larger populations and a longer follow-up period.

## 5. Conclusions

Obstructive sleep apnea signs and greater airflow limitation were associated with endothelial dysfunction, regardless of the severity of the coronary artery disease. Endothelial dysfunction might be one of the mechanisms explaining the relationship between sleep apnea and cardiovascular diseases.

## Figures and Tables

**Table 1:** Demographics, comorbidities and pharmacotherapy in patients with and without endothelial dysfunction.

	Normal Endothelial Function *n* = 70	Endothelial Dysfunction * n = 43	*p* Value
Age [years]	64.8 ± 11.8	64.1 + 12.8	0.77
Male	73%	81%	0.21
Previous MI	53%	37%	0.08
Previous PCI	56%	42%	0.11
Previous CABG	14%	9.3%	0.32
Previous stroke	8.6%	9.3%	0.57
Peripheral artery disease	11%	16%	0.32
Hypertension	80%	79%	0.54
Diabetes	31%	42%	0.18
Chronic kidney disease	5.7%	9.3%	0.36
Hypercholesterolemia	33%	30%	0.47
COPD	4.3%	7.0%	0.41
Asthma	1.4%	2.3%	0.62
Atrial fibrillation	7.1%	19%	0.06
Smoking	24%	21%	0.43
Chest pain	81%	79%	0.76
BMI [kg/m^2^]	**28.5 ± 4.3**	**30.5 ± 6.1**	**0.049**
Waist circumference [cm]	80 ± 43	83 ± 48	0.75
Systolic blood pressure [mm Hg]	130 ± 20	130 ± 20	0.64
Diastolic blood pressure [mm Hg]	70 ± 10	71 ± 10	0.71
Heart rate [beats per minute]	65 ± 14	65 ± 10	0.59
Pharmacotherapy			
Aspirin	93%	84%	0.11
P2Y12 inhibitor	40%	35%	0.37
β-adrenergic receptor inhibitor	84%	84%	0.57
ACE inhibitor	74%	61%	0.09
ARB	14%	19%	0.36
Calcium blocker	20%	14%	0.29
MRA	13%	7%	0.26
Thiazide diuretics	13%	16%	0.4
Loop diuretics	**11%**	**35%**	**0.003**
Statins	80%	79%	0.54
Fibrates	2.9%	4.7%	0.49
Sulphonylureas	13%	4.7%	0.13
Metformin	19%	32%	0.07
Insulin	5.7%	11%	0.22
LABA	4.3%	2.3%	0.51
LAMA	1.4%	0%	0.62
Inhaled corticosteroids	1.4%	0%	0.62
SABA			
SAMA	1.4%	2.3%	0.62
Vitamin K antagonists	0%	4.7%	0.14
NOAC	2.9%	7%	0.28
α-adrenergic receptor inhibitor	1.4%	7%	0.15
Nitrates	2.9%	7%	0.28

ACE—angiotensin-converting enzyme; ARB—angiotensin receptor blockers; BMI—body mass index; CABG—coronary artery bypass graft; COPD—chronic obstructive pulmonary disease; LABA—long-acting beta-agonists; LAMA—long-acting muscarinic antagonists; MI—myocardial infarction; MRA—mineralocorticoid receptor antagonist; NOAC—non-vitamin K antagonist oral anticoagulant; PCI—percutaneous coronary intervention; SAMA—short-acting muscarinic-antagonist * Endothelial dysfunction diagnosed in patients with reactive hyperemia index less than 1.67. Data are presented as percentages and as mean ± standard deviation. The bold text indicates *p* values < 0.05.

**Table 2:** Coronary artery disease, echocardiography data and basic laboratory findings.

	Normal Endothelial Function *n* = 70	Endothelial Dysfunction * n = 43	*p* Value
Coronary angiography			
No significant coronary artery disease	13%	23%	0.53
Single-vessel disease	19%	19%	
Two-vessel disease	31%	28%	
Three-vessel disease	37%	30%	
Echocardiography			
Left ventricle diastolic diameter [cm]	5.03 ± 0.64	5.12 ± 0.57	0.44
Intraventricular septum [cm]	**1.14 ± 0.22**	**1.21 ± 0.15**	**0.049**
Left atrium [cm]	4.09 ± 0.49	4.31 ± 0.64	0.05
Right ventricle [cm]	3.0 ± 0.37	3.0 ± 0.38	0.98
Left ventricle ejection fraction [%]	53.09 ± 9.23	52.70 ± 9.23	0.83
Posterior wall thickness [cm]	**0.95 ± 0.17**	**1.05 ± 0.14**	**0.004**
Ascending aorta diameter [cm]	3.36 ± 0.36	3.45 ± 0.4	0.31
E’ medial	6.89 ± 1.69	6.22 ± 1.84	0.09
E’ lateral	8.46 ± 2.58	8.05 ± 2.32	0.46
E	73.23 ± 18.2	74.94 ± 18.1	0.67
A	80.20 ± 17.8	87.46 ± 18.2	0.07
E/A	0.93 ± 0.27	0.95 ± 0.50	0.87
Deceleration time [ms]	211 ± 65	223.5 ± 66	0.5
TRPG [mmHg]	22.05 ± 5.1	23.96 ± 9.8	0.36
TAPSE [mm]	23.40 ± 4.1	23.95 ± 4.3	0.5
Laboratory tests			
GFR [mL/min/m^2^]	60 ± 4.2	60 ± 13.2	0.32
CRP [mg/L]	1.5 ± 1.6	1.9 ± 3.7	0.15
NT pro BNP [pg/mL]	539.42 ± 1406	467.83 ± 791	0.76

CRP—C-reactive protein; GFR—glomerular filtration rate; NT-proBNP—N-terminal (NT)-prohormone BNP; TAPSE—tricuspid annulus plane systolic excursion; TRPG—tricuspid regurgitation peak gradient * Endothelial dysfunction diagnosed in patients with reactive hyperemia index less than 1.67. Data are presented as percentages and as mean ± standard deviation. The bold text indicates *p* values < 0.05.

**Table 3:** Polygraphy and pulmonary function tests data.

	Normal Endothelial Function *n* = 70	Endothelial Dysfunction * n = 43	*p* Value
Epworth sleepiness scale	5.36 ± 3.9	6.42 ± 5	0.24
PAT respiratory disturbance index	**14.8 ± 13.7**	**27.32 ± 22.9**	**0.001**
PAT apnea and hypopnea index	**10.30 ± 12.8**	**24.6 ± 24.7**	**<0.001**
Oxygen desaturation index	**5.23 ± 7.0**	**13.73 ± 17.2**	**0.002**
CAT questionnaire	10.03 ± 5.8	10.93 ± 7.3	0.47
FEV_1_ (L)	2.64 ± 0.8	2.46 ± 0.8	0.26
FEV_1_% pred.	**89.01 ± 18.4**	**81.17 ± 19.6**	**0.04**
FVC (L)	3.85 ± 1	3.65 ± 1	0.36
FVC% pred.	100.02 ± 17	93.18 ± 19	0.05
FEV_1_%FVC	68.56 ± 8	67.55 ± 10	0.55
PEF (L/S)	7.3 ± 2.3	7.0 ± 2.3	0.5
PEF% pred.	93.36 ± 27	89.35 ± 24	0.44
TLC (L)	6.76 ± 1.4	6.87 ± 1.3	0.7
TLC% pred.	110.17 ± 13	106.84 ± 13	0.21
RV (L)	2.86 ± 0.6	3.01 ± 0.8	0.3
RV% pred.	125.02 ± 25	126.6 ± 29	0.77
RV/TLC	43.24 ± 9	44.06 ± 8	0.65
RV/TLC% pred.	108.21 ± 18	111.98 ± 19	0.31
FRC (L)	3.97 ± 1	4.02 ± 1	0.75
FRC% pred.	121.80 ± 26	117.97 ± 25	0.47

CAT—chronic obstructive pulmonary disease assessment test; FEV1—forced expiratory volume in one second; FRC—functional residual capacity; FVC—forced vital capacity; PAT—peripheral arterial tonometry; PEF—peak expiratory flow; pred.—predictive value; RV—residual volume; TLC—total lung capacity; VC—vital capacity * Endothelial dysfunction diagnosed in patients with reactive hyperemia index less than 1.67. Data are presented as mean ± standard deviation. The bold text indicates *p* values < 0.05.

**Table 4:** Multivariate regression analysis of reactive hyperemia index determinants *.

	Β-Coefficient	95% CI	*p* Value
BMI	0.003	−0.024–0.024	0.98
Previous MI	−0.036	−0.277–0.193	0.72
Atrial fibrillation	0.021	−0.313–0.387	0.83
FEV_1_% pred.	0.219	0.001–0.013	0.03
pAHI	−0.313	−0.021–0.004	0.003

BMI—body mass index; FEV1—forced expiratory volume in one second; pAHI—peripheral arterial tonometry apnea and hypopnea index; MI—myocardial infarction. * The model included variables showing at least moderate association (*p* < 0.1) with the endothelial dysfunction.

## Data Availability

All data will be available under correspondence via e-mail address for three years following the publication after a request that is justifiable.
